# Role of CD73 in renal sympathetic neurotransmission in the mouse kidney

**DOI:** 10.1002/phy2.57

**Published:** 2013-08-22

**Authors:** Edwin K Jackson, Dongmei Cheng, Zaichuan Mi, Jonathan D Verrier, Keri Janesko-Feldman, Patrick M Kochanek

**Affiliations:** 1 Department of Pharmacology and Chemical Biology, University of Pittsburgh School of MedicinePittsburgh, Pennsylvania; 2 Safar Center for Resuscitation Research, University of Pittsburgh School of MedicinePittsburgh, Pennsylvania; 3 Department of Critical Care Medicine, University of Pittsburgh School of MedicinePittsburgh, Pennsylvania

**Keywords:** 5′-AMP, A_1_ receptor, adenosine, CD73, ecto-5′-nucleotidase, norepinephrine, renal sympathetic neurotransmission

## Abstract

Adenosine formed during renal sympathetic nerve stimulation (RSNS) enhances, by activating A_1_ receptors, the postjunctional effects of released norepinephrine and participates in renal sympathetic neurotransmission. Because in many cell types CD73 (ecto-5′-nucleotidase) is important for the conversion of 5′-AMP to adenosine, we investigated whether CD73 is necessary for normal renal sympathetic neurotransmission. In isolated kidneys from CD73 wild-type mice (CD73+/+; *n* = 17) perfused at a constant rate with Tyrode's solution, RSNS increased perfusion pressure by 17 ± 4, 36 ± 8, and 44 ± 10 mm Hg at 3, 5, and 7 Hz, respectively. Similar responses were elicited from kidneys isolated from CD73 knockout mice (CD73−/−; *n* = 13; 28 ± 11, 43 ± 10, and 44 ± 10 mm Hg at 3, 5, and 7 Hz, respectively); and a high concentration (100 μmol/L) of α,β-methyleneadenosine 5′-diphosphate (CD73 inhibitor) did not alter responses to RSNS in C57BL/6 mouse kidneys (*n* = 5; 21 ± 5, 36 ± 8, and 43 ± 9 at 3, 5, and 7 Hz, respectively). Measurements of renal venous adenosine and inosine (adenosine metabolite) by liquid chromatography-tandem mass spectrometry demonstrated that the metabolism of exogenous 5′-AMP to adenosine and inosine was similar in CD73−/− versus CD73+/+ kidneys. A_1_ receptor mRNA expression was increased in CD73−/− kidneys, and 2-chloro-N^6^-cyclopentyladenosine (0.1 μmol/L; A_1_ receptor agonist) enhanced renovascular responses to norepinephrine more in CD73−/− versus CD73+/+ kidneys. We conclude that CD73 is not essential for renal sympathetic neurotransmission because in the absence of renal CD73 other enzymes metabolize 5′-AMP to adenosine and because of compensatory upregulation of postjunctional coincident signaling between norepinephrine and adenosine.

## Introduction

Studies in isolated, perfused rat kidneys show that pharmacological blockade of A_1_ receptors attenuates renal vasoconstriction induced by renal sympathetic nerve simulation (RSNS) without changing renal spillover of norepinephrine (Jackson et al. 2012b). Also, selective activation of A_1_ receptors with 2-chloro-N^6^-cyclopentyladenosine (CCPA) enhances renal vasoconstriction induced by norepinephrine in the rat kidney. This latter effect is blocked by inhibition of phospholipase C, protein kinase C, c-src, phosphatidylinositol 3-kinase, and 3-phosphoinositide-dependent protein kinase-1 (Jackson et al. 2012b). Consistent with these findings is the very recent report that renal sympathetic neurotransmission is impaired in kidneys isolated from mice null for the A_1_ receptor (Jackson et al. 2012a). These studies provide strong evidence for the concept that adenosine formed during RSNS enhances the postjunctional effects of released norepinephrine via coincident signaling and contributes to renal sympathetic neurotransmission (Jackson et al. 2012b). Likely, the coincident signaling pathway involves: phospholipase C → protein kinase C → c-src → phosphatidylinositol 3-kinase → 3-phosphoinositide-dependent protein kinase-1 (Jackson et al. 2012b).

Although studies to date provide compelling evidence for the involvement of adenosine in renal sympathetic neurotransmission, it is unclear as to the enzymatic machinery that mediates the formation of adenosine in the renal neuroeffector junction. In this regard, elegant experiments by Westfall and coworkers in the guinea pig vas deferens show that adenosine can be formed in the neuroeffector junction due to release of the cotransmitter ATP which can be rapidly metabolized by releasable nucleotidases (which undergo exocytosis along with ATP) to adenosine (the Westfall mechanism: releasable enzymes mediate the pathway ATP → ADP → 5′-AMP → adenosine) (Westfall et al. 2002).

CD73, also called ecto-5′-nucleotidase, is an ecto-enzyme that in many organ systems is critical to the conversion of 5′-AMP to adenosine in the extracellular compartment (Deaglio and Robson 2011). Indeed, in the kidney CD73 participates in the production of adenosine that mediates tubuloglomerular feedback (Castrop et al. 2004), provides renoprotective adenosine that renders the kidneys more tolerate to acute ischemia/reperfusion (Grenz et al. 2007), and produces adenosine in response to chronic exposure to angiotensin II leading to A_2B_ receptor-induced chronic renal disease and hypertension (Zhang et al. 2013). Therefore, reasoning by induction supports the hypothesis that CD73 is involved in the formation of adenosine by other renal physiological mechanisms such as renal sympathetic neurotransmission. Moreover, because CD73 is a glycosyl phosphatidylinositol (GPI)-anchored enzyme and because GPI-anchored enzymes are well known to be released as soluble enzymes (Butikofer et al. 2001), CD73 could be in part the releasable ecto-AMPase discussed by Westfall, a conclusion reinforced by the observation that in the guinea pig vas deferens inhibition of CD73 blocks the activity of the AMPase released by sympathetic nerve stimulation (Westfall et al. 2002).

Because of the strong rationale suggesting a role for CD73 in renal sympathetic neurotransmission, we initiated the present study to test this concept by employing several investigative tools available in our lab: (1) a technique for examining vascular responses to RSNS in the isolated, perfused mouse kidney (Ren et al. 2008); (2) a method for measuring small quantities of purines using high performance liquid chromatography-tandem mass spectrometry (Jackson et al. 2009), and (3) the maintenance of a breeding colony of CD73 knockout mice (Jackson et al. 2011).

## Methods

### Animals

Male and female CD73−/− mice (Castrop et al. 2004) (C57BL/6 x J129 background) and wild-type litter mates (CD73+/+ mice) were bred and genotyped (Castrop et al. 2004) at the University of Pittsburgh. Mice used for experiments were 10 to12 weeks of age with similar numbers of male and female mice. C57BL/6 mice were obtained from Taconic Farms (Germantown, NY), and because no gender differences were noted in the experiments with CD73−/− and +/+ mice, studies with C57BL/6 were conducted only in male animals. Animals were housed at the University of Pittsburgh Animal Facility and fed Pro Lab RHM 3000 rodent diet (PMI Feeds, St. Louis, MO). The Institutional Animal Care and Use Committee approved all procedures. The investigation conforms to the *Guide for the Care and Use of Laboratory Animals* published by the US National Institutes of Health (NIH Publication No. 85-23, revised 1996).

### Drugs

α,β-Methyleneadenosine 5′-diphosphate [AMPCP; CD73 inhibitor (Zimmermann 1992)] and 2-chloro-N^6^-cyclopentyladenosine [CCPA; a highly selective A_1_ receptor agonist (Jacobson and Knutsen 2001)] were obtained from Sigma-Aldrich (St. Louis, MO).

### Isolated, perfused mouse kidney

Mouse kidneys were isolated and perfused at a constant rate (1.5 ml/min) with Tyrode's solution as previously described by us (Ren et al. 2008). Kidneys were allowed to stabilize for 2 h before initiating the protocols described below.

### Renal sympathetic nerve stimulation

Renal sympathetic nerve stimulation (RSNS) was accomplished by placing a platinum bipolar electrode around the renal artery close to the kidney and connecting the electrode to a Grass stimulator (model SD9E; Grass Instruments, Quincy, MA) as previously described by us (Ren et al. 2008). The tissues around the electrode were kept moist with Tyrode's solution and the stimulation parameters were biphasic pulses; 1-ms pulse duration; 35 V at indicated frequencies.

### Analysis of purines

5′-AMP, adenosine, and inosine were quantified using ultra-performance liquid chromatography-tandem mass spectrometry (LC-MS/MS) as previously described by us (Ren et al. 2008).

### Real-time PCR for A_1_ receptor mRNA

Total RNA was isolated from kidneys obtained from both CD73+/+ and −/− mice using Trizol (Life Technologies, Carlsbad, CA) according to the manufacturer's instructions. Using gene-specific primers for the adenosine A_1_ receptor (Qiagen, Gaithersburg, MD, catalog number QT00301119) and glyceraldehyde-3-phosphate dehydrogenase (GAPDH) (Qiagen, catalog number QT01658692) semi-quantitative real-time PCR was performed using an Applied Biosystems 7900HT Real-Time PCR System (Carlsbad, CA). There were four samples (all from separate mice) per genotype and each sample was run in duplicate for each primer pair tested. The duplicates were averaged and the A_1_ receptor mRNA expression was normalized for GAPDH levels. Comparisons between genotypes was performed using the 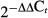 method (Livak and Schmittgen 2001).

### Protocol 1

Perfused kidneys from CD73+/+ (*n* = 17) or CD73−/− (*n* = 13) mice were subjected to RSNS at increasing frequencies [0 (basal), 3, 5, and 7 Hz] for 5 min at each frequency and a venous perfusate sample was collected between 4 and 5 min during each stimulation period. Samples were heat inactivated to prevent enzymatic degradation of purines and stored at −80°C until assayed by LC-MS/MS. Next, after a rest period of 20 min, norepinephrine (NE) was infused at increasing concentrations (50, 100, and 150 nmol/L; final concentration in perfusate) for 5 min at each concentration. After another rest period of 20 min, kidneys were infused with CCPA (100 nmol/L; final concentration in perfusate). While maintaining the infusion of CCPA, the concentration response to NE was repeated.

### Protocol 2

In a separate group of perfused kidneys from C57BL/6 mice, AMPCP (100 μmol/L; final concentration in perfusate; *n* = 5) was added to the Tyrode's solution beginning 1 hour into the 2-hour rest period; whereas in some kidneys (*n* = 16) no inhibitor was added. Kidneys were subjected to RSNS at increasing frequencies [0 (basal), 3, 5, and 7 Hz] for 5 min at each frequency.

### Protocol 3

This protocol consisted of 16 CD73+/+ kidneys and 16 CD73−/− kidneys. AMPCP (100 μmol/L; final concentration in perfusate) was added to the perfusate of half of the CD73+/+ and half of the CD73−/− kidneys beginning 1 hour into the 2-hour rest period. A basal renal venous sample was collected, then 5′-AMP (10 μmol/L) was added to the perfusate and 5 min later another renal venous sample was collected. Samples were heat inactivated to prevent enzymatic degradation of purines and stored at −80°C until assayed by LC-MS/MS.

### Statistics

Data were analyzed by nested two-factor or three-factor analysis of variance ANOVA. The criterion of significance was *P* < 0.05. All values in text and figures are means ± SEM.

## Results

**Figure 1:**
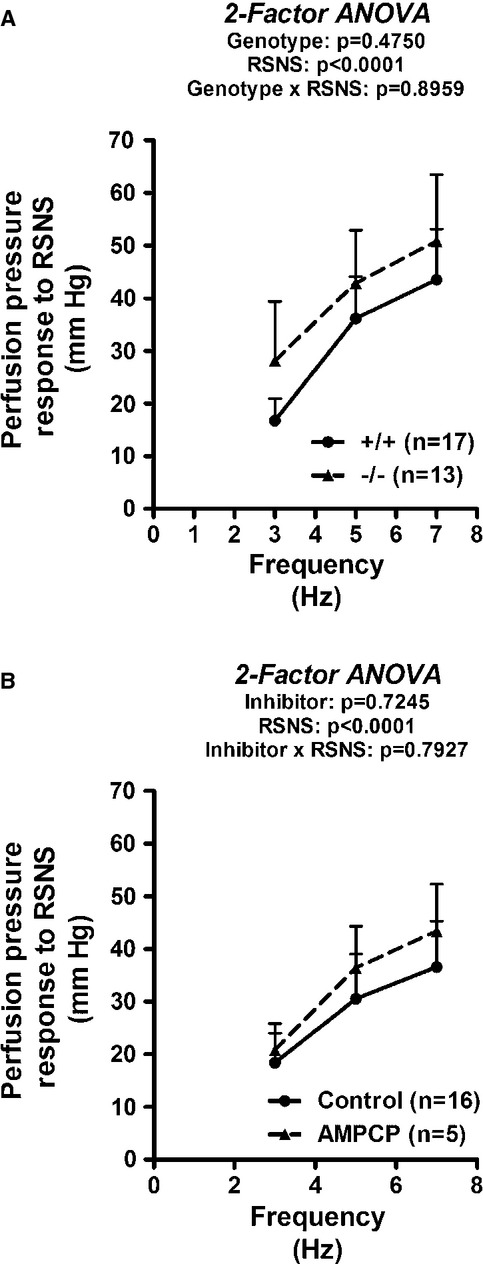
Line graphs show effects of renal sympathetic nerve stimulation (RSNS) on changes in renal perfusion pressure. (A) Compares RSNS-induced responses in CD73+/+ versus CD73−/− kidneys. Basal perfusion pressures were 42 ± 2 and 39 ± 1 mm Hg in CD73+/+ versus CD73−/− kidneys, respectively. (B) Compares RSNS-induced responses in untreated (Control) versus AMPCP-treated (AMPCP; 100 μmol/L) kidneys from C57BL/J mice. Basal perfusion pressures were 41 ± 2 and 40 ± 3 in Control versus AMPCP kidneys, respectively. *P*-values in panels are from nested two-factor analysis of variance (ANOVA). All values represent means ± SEM.

As shown in Fig. 1A, RSNS induced a frequency-dependent increase (*P* < 0.0001) in perfusion pressure; however, there was neither a significant overall effect of CD73 genotype on RSNS-induced responses (*P* = 0.4750) nor a significant interaction between CD73 genotype and responses to RSNS (*P* = 0.8959). We also examined the effects of a high concentration of AMPCP (100 μmol/L) on responses to RSNS. Again, RSNS induced a frequency-dependent increase (*P* < 0.0001) in perfusion pressure (Fig. 1B); and again there was neither a significant overall effect of CD73 inhibition on RSNS-induced responses (*P* = 0.7245) nor a significant interaction between CD73 inhibition and responses to RSNS (*P* = 0.7927).

**Figure 2:**
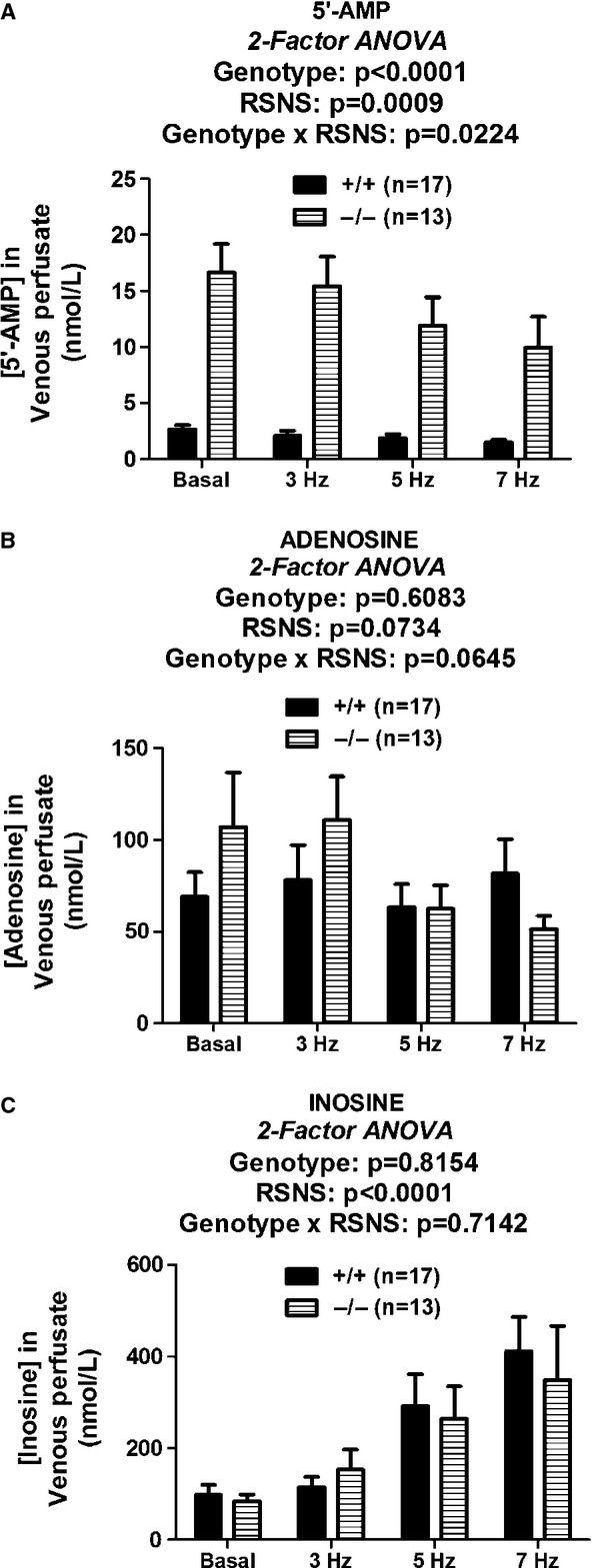
Bar graphs illustrate renal venous levels of 5′-AMP (A), adenosine (B) and inosine (C) before and during renal sympathetic nerve stimulation (RSNS) at the indicated frequencies in CD73+/+ versus CD73−/− kidneys. *P*-values in panels are from nested two-factor analysis of variance. All values represent means ± SEM.

As shown in Fig. 2A, basal renal venous levels of 5′-AMP were approximately sixfold higher in CD73−/− compared with CD73+/+ kidneys (*P* < 0.0001); and RSNS caused a frequency-related reduction in renal venous levels of 5′-AMP (*P* = 0.0009). Basal renal venous adenosine levels were similar in CD73−/− versus CD73+/+ kidneys (*P* = 0.6083) and were not altered by RSNS (*P* = 0.0734) (Fig. 2B). Basal inosine levels were similar in CD73−/− versus CD73+/+ kidneys (*P* = 0.8154) and were similarly increased by RSNS in CD73−/− and CD73+/+ kidneys (*P* < 0.0001) (Fig. 2C).

**Figure 3:**
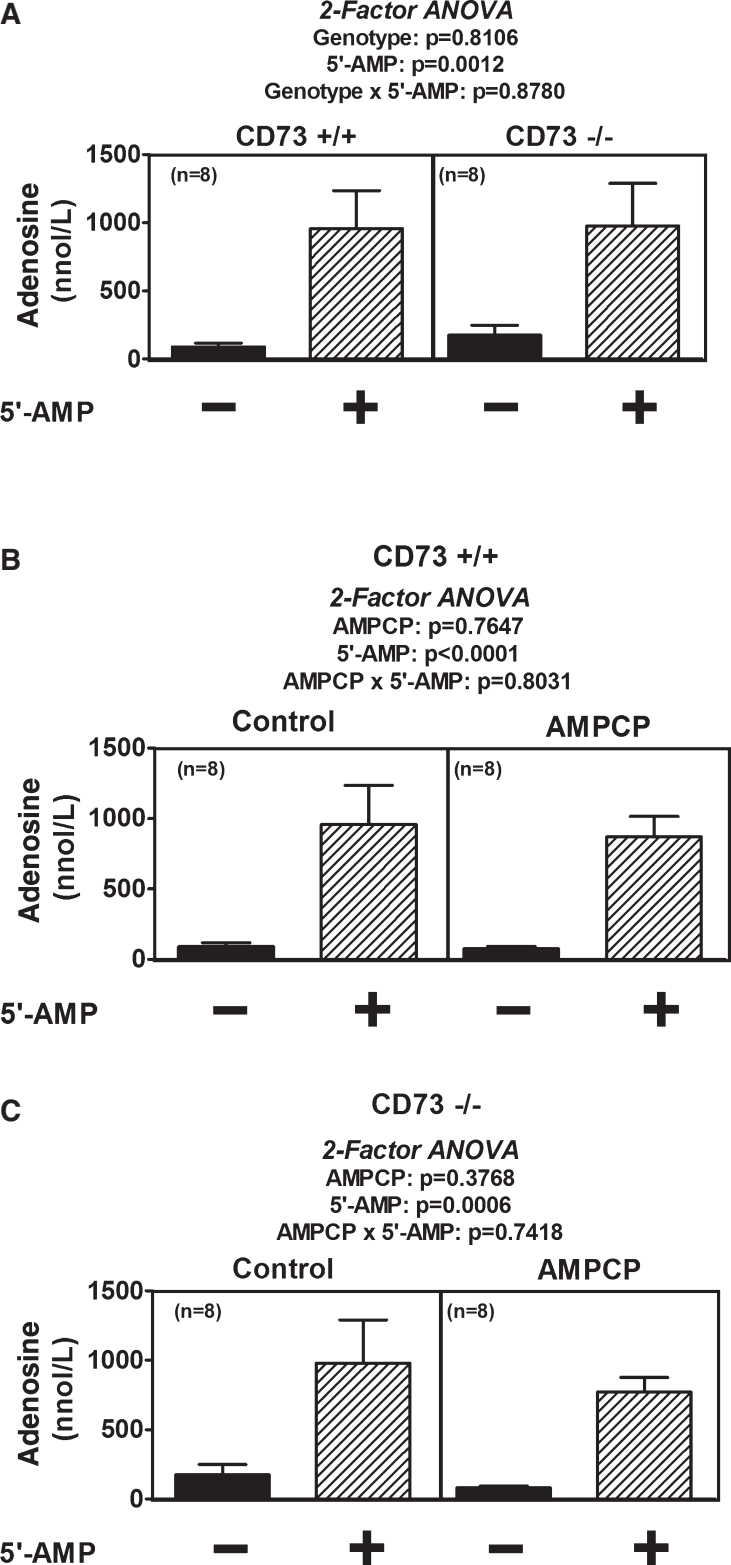
Bar graphs show renal venous levels of adenosine before and after addition of 5′-AMP (10 μmol/L) to the perfusate. (A) Compares 5′-AMP-induced adenosine in CD73+/+ versus CD73−/− kidneys. (B and C) Compare 5′-AMP-induced adenosine in the absence and presence of AMPCP (100 μmol/L) in CD73+/+ and CD73−/− kidneys, respectively. *P*-values in panels are from nested two-factor analysis of variance. All values represent means ± SEM.

**Figure 4:**
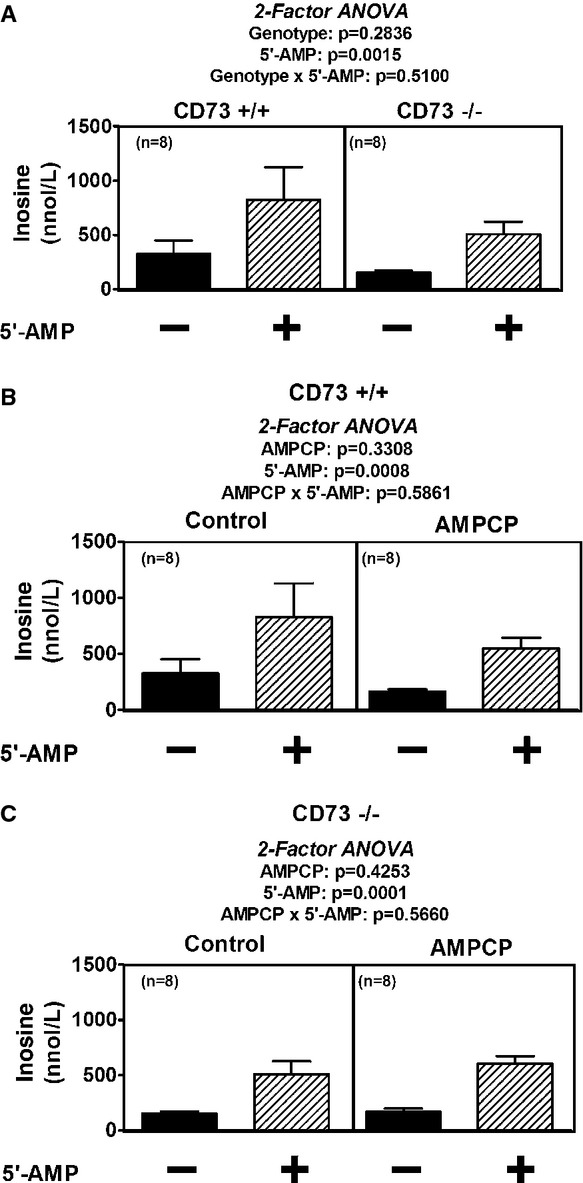
Bar graphs illustrate renal venous levels of inosine before and after addition of 5′-AMP (10 μmol/L) to the perfusate. (A) Compares 5′-AMP-induced inosine in CD73+/+ versus CD73−/− kidneys. (B and C) Compare 5′-AMP-induced inosine in the absence and presence of AMPCP (100 μmol/L) in CD73+/+ and CD73−/− kidneys, respectively. *P*-values in panels are from nested two-factor analysis of variance. All values represent means ± SEM.

Infusions of 5′-AMP caused a significant increase in renal venous adenosine levels (*P* = 0.0012); however, the increase was similar in CD73−/− versus +/+ kidneys (*P* = 0.8780) (Fig. 3A). AMPCP (100 μmol/L) did not inhibit the 5′-AMP-induced production of adenosine in either CD73+/+ kidneys (*P* = 0.8031) (Fig. 3B) or CD73−/− kidneys (*P* = 0.7418) (Fig. 3C). Likewise, infusions of 5′-AMP caused a significant increase in renal venous inosine levels (*P* = 0.0015); however, the increase was similar in CD73−/− versus +/+ kidneys (*P* = 0.5100) (Fig. 4A). AMPCP (100 μmol/L) did not inhibit the 5′-AMP-induced production of inosine in either CD73+/+ kidneys (*P* = 0.5861) (Fig. 4B) or CD73−/− kidneys (*P* = 0.5660) (Fig. 4C).

**Figure 5:**
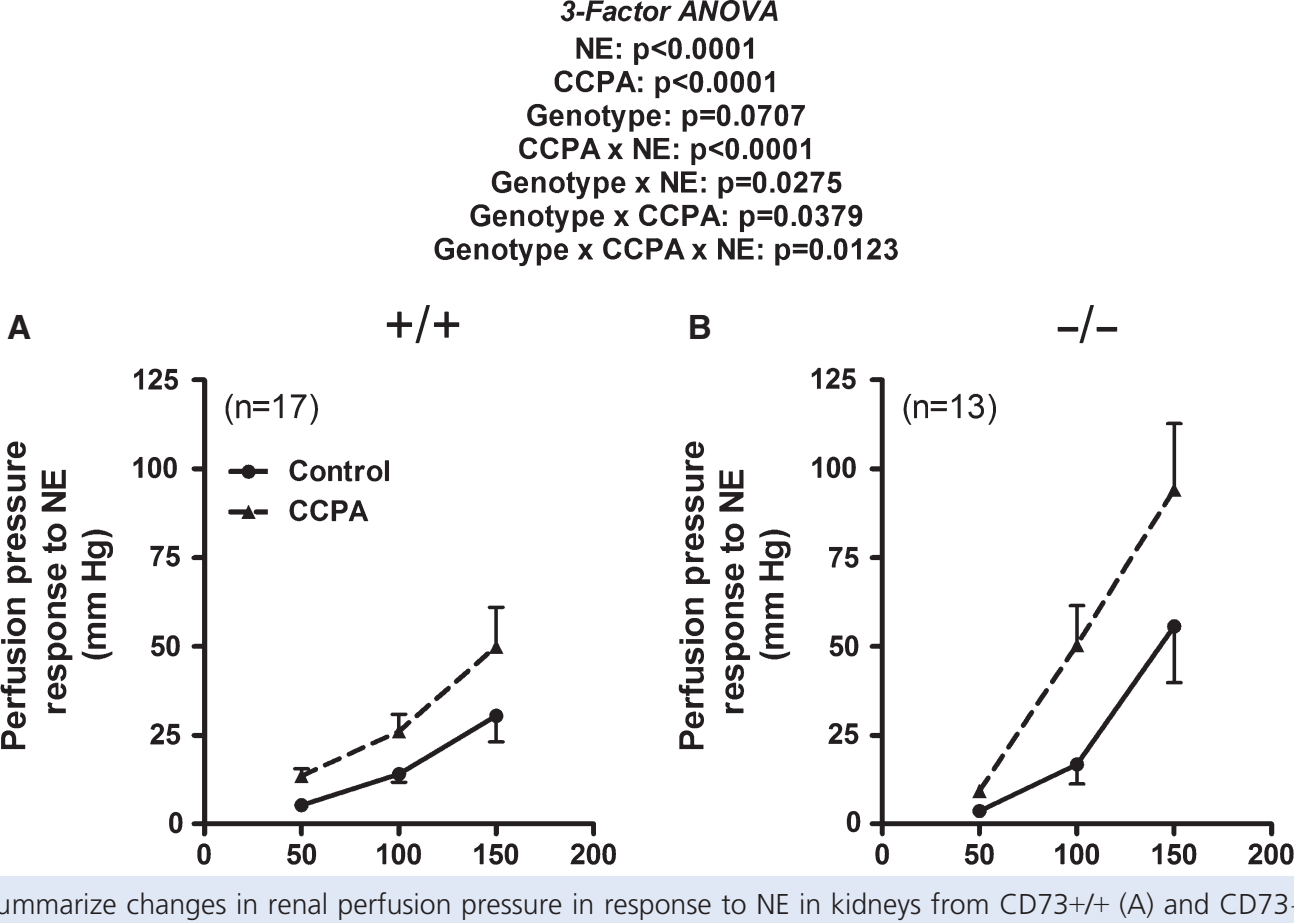
Line graphs summarize changes in renal perfusion pressure in response to NE in kidneys from CD73+/+ (A) and CD73−/− (B) mice before and during treatment with 2-chloro-N^6^-cyclopentyladenosine (CCPA; 100 nmol/L). In CD73+/+ kidneys, basal perfusion pressures were 40 ± 2 and 44 ± 2 mm Hg before and during CCPA, respectively. In CD73−/− kidneys, basal perfusion pressures were 38 ± 1 and 39 ± 1 mm Hg before and during CCPA, respectively. *P*-values in panels are from nested three-factor analysis of variance. All values represent means ± SEM.

Norepinephrine (NE) caused a significant and concentration-dependent increase in renal perfusion pressure that was significantly (*P* < 0.0001) augmented by CCPA (0.1 μmol/L) in both CD73+/+ (Fig. 5A) and CD73−/− (Fig. 5B) kidneys. Moreover, three-factor ANOVA indicated a significant three-way interaction (*P* = 0.0123) involving genotype, CCPA, and NE which was due to the fact that CCPA augmented renovascular responses to NE more in CD73−/− compared with CD73+/+ kidneys. Real-time PCR revealed that the expression level of A_1_ receptor mRNA was three times higher in CD73−/− versus CD73+/+ kidneys (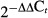: CD73+/+, 1.24 ± 0.29; CD73−/−, 3.71 ± 1.41; *P* < 0.05, Wilcoxon rank-sum test).

## Discussion

Our previously published work demonstrates that in isolated, perfused rat kidneys, pharmacological antagonism of A_1_ receptors attenuates renovascular responses to RSNS without affecting RSNS-induced release of NE (Jackson et al. 2012b). Subsequent experiments in isolated, perfused kidneys from A_1_ receptor null mice confirm the concept that renal sympathetic neurotransmission relies upon A_1_ receptor signaling to provide for a full response to RSNS (Jackson et al. 2012a). The underlying mechanism appears to be postjunctional coincident signaling between A_1_ receptors and α-adrenoceptors because selective activation of A_1_ receptors with CCPA augments renal vasoconstriction induced by NE, a phenomenon that is blocked by selective A_1_ receptor antagonists and knockout of A_1_ receptors (Jackson et al. 2012a,b). Consistent with the notion that A_1_ receptors modulate vasoconstriction in the renal vasculature is the recent finding by Li and coworkers showing that targeted deletion of vascular A_1_ receptors abolishes vasoconstrictor responses to adenosine in afferent arterioles (Li et al. 2012).

How is adenosine formed in the renal neuroeffector junction? Previous experiments in mouse kidneys show that RSNS causes a frequency-dependent release of inosine (Ren et al. 2008; Jackson et al. 2012a), the immediate downstream metabolite of adenosine, into the renal venous perfusate. Although RSNS increases renal venous inosine, RSNS does not increase renal venous adenosine and actually reduces renal venous 5′-AMP (Ren et al. 2008; Jackson et al. 2012a). These observations are consistent with the hypothesis proposed by Westfall and coworkers that adenosine is formed in the sympathetic neuroeffector junction due to the rapid metabolism of the sympathetic cotransmitter ATP by soluble nucleotidases which appear to be coreleased with ATP (Westfall et al. 2002). Release of AMPases by RSNS would explain the reduction in 5′-AMP reaching the renal venous effluent, and the formation of adenosine in the neuroeffector junction would explain the increase in inosine, but not adenosine, because adenosine in the neuroeffector junction would likely be metabolized to inosine during the passage from its site of production in the neuroeffector junction to the renal vein. Thus, a body of evidence is converging on the concept that RSNS induces the formation of adenosine in renal sympathetic neuroeffector junctions, and adenosine, via A_1_ receptors, enhances renal sympathetic neurotransmission by coincident signaling with NE.

A priori it is likely that CD73 (ecto-5′-nucleotidase) is involved in the formation of adenosine in the renal sympathetic neuroeffector junction. Indeed, in the guinea pig vas deferens, the AMPase released by sympathetic nerve stimulation is blocked by AMPCP (Mihaylova-Todorova et al. 2002), a drug that is a potent (Ki = 0.0006 μmol/L) CD73 inhibitor (Naito and Lowenstein 1985). This suggests that CD73 is involved in adenosine formation in the sympathetic neuroeffector junction of the guinea pig vas deferens; however, whether this is the case in the renal sympathetic neuroeffector junction is unknown.

The present study shows that neither complete knockout of CD73 nor pharmacological inhibition with AMPCP (using an AMPCP concentration approximately 166,000-fold greater than the Ki of AMPCP for CD73) attenuates RSNS-induced renal vasoconstriction. These findings definitively show that CD73 is not necessary for normal renal sympathetic neurotransmission.

How is it that the A_1_ receptor is required for normal renal sympathetic neurotransmission and CD73 is a major adenosine-generating enzyme, yet CD73 is not required for normal renal sympathetic neurotransmission? There are at least three possible (and nonmutually exclusive) reasons for the fact that CD73 is not a necessary participant in renal neurotransmission. First, our studies show that there is a remarkable accumulation (sixfold) of endogenous 5′-AMP in CD73−/− kidneys. Likely this is because of the bottleneck in 5′-AMP metabolism that is created by deleting CD73. Important recent studies by Rittiner et al. (2012) show that 5′-AMP per se is a full agonist at A_1_ receptors. Therefore, one reason that CD73 is not required for renal sympathetic neurotransmission may be that the accumulated 5′-AMP can activate postjunctional A_1_ receptors. In other words, when CD73 is blocked, 5′-AMP substitutes for adenosine in the neuroeffector junction. Second, deleting or blocking CD73 does not seem to impair renal adenosine production (at least as assessed by adenosine levels in the renal venous outflow). For example, RSNS increases renal venous inosine similarly in CD73−/− versus +/+ kidneys, and exogenous 5′-AMP increases renal venous adenosine and inosine similarly in CD73+/+, AMPCP-treated CD73+/+, CD73−/−, and AMPCP-treated CD73−/− kidneys. Most likely when 5′-AMP accumulates behind the CD73 block, other “shunt” pathways are available that generate adenosine. An important caveat, however, is that CD73 may be critical for adenosine formation in some compartments of the kidney that are not reflected by renal venous sampling. Third, with chronic deletion of CD73 the mechanism of coincident signaling becomes upregulated such that activation of the A_1_ receptor causes an even greater enhancement of α-adrenoceptor-induced signaling and vasoconstriction. This concept is supported by the observation that A_1_ receptor mRNA is elevated threefold in CD73−/− kidneys suggesting upregulation of A_1_ receptors. We attempted to measure A_1_ receptor protein using three different commercial antibodies to rat A_1_ receptors, but all three did not cross-react with the mouse receptor. Nonetheless, it is likely that the increased ability of A_1_ receptor agonists to augment the effects of α-adrenoceptor-induced signaling and vasoconstriction is due in part to A_1_ receptor upregulation and possibly also to upregulation of postreceptor signaling.

In summary, the present work firmly establishes that CD73 is not necessary for adenosine biosynthesis in those renal compartments in communication with the renal venous outflow and that CD73 is not necessary for normal renal sympathetic neurotransmission. These findings are quite important for several reasons. First, our results show that blockade of CD73 can have, at least in some organ systems, minimal effects on adenosine production because of accumulation of 5′-AMP and shunting of this substrate down alternative enzymatic pathways that can generate adenosine from 5′-AMP. This reinforces the importance of investigating nontraditional pathways of adenosine formation from 5′-AMP and thus informs and guides future studies in this regard. These finding also underscore the general principle of being mindful of the fact that there is an increasing body of evidence regarding the role of alternate pathways in complex biological systems that are engaged when a targeted pathway is obstructed genetically or pharmacologically. For example, with reference to the present study, our general conclusion is that lack of an effect of CD73 inhibition does not necessarily imply lack of a role for adenosine or adenosine receptors. Second, development of therapeutic strategies to manipulate adenosine production would be, in many cases, more productive if aimed at upstream components of the adenosine-generating pathway, for example, CD39 that mediates the metabolism of ATP to ADP. This would prevent the formation of both 5′-AMP (A_1_ receptor agonist) and adenosine (agonist at all adenosine receptors). Finally, CD73 is an important target for drug development, and inhibitors of CD73 may find applications for the treatment of several diseases. For example, CD73 inhibitors may be beneficial in cancer by decreasing adenosine production by regulatory T cells and thus augmenting the ability of effector T cells to inhibit tumor growth (Mandapathil et al. 2010a,b; Yegutkin et al. 2011) and may be beneficial in chronic kidney disease and angiotensin II-mediated hypertension (Zhang et al. 2013). The present findings indicate that although chronic CD73 inhibitors would not cause impairment of renal sympathetic neurotransmission, such inhibitors could augment coincident signaling in the renal vasculature with unknown consequences.
